# The pretreatment of corn stover with *Gloeophyllum trabeum* KU-41 for enzymatic hydrolysis

**DOI:** 10.1186/1754-6834-5-28

**Published:** 2012-05-04

**Authors:** Ziqing Gao, Toshio Mori, Ryuichiro Kondo

**Affiliations:** 1 Department of Agro-Environmental Sciences, Faculty of Agriculture, Kyushu, University, 6-10-1 Hakozaki, Higashi-ku, Fukuoka, 812-8581, Japan

**Keywords:** Brown-rot fungus, *Gloeophyllum trabeum*, Corn stover, Bio-ethanol

## Abstract

**Background:**

Pretreatment is an essential step in the enzymatic hydrolysis of biomass for bio-ethanol production. The dominant concern in this step is how to decrease the high cost of pretreatment while achieving a high sugar yield. Fungal pretreatment of biomass was previously reported to be effective, with the advantage of having a low energy requirement and requiring no application of additional chemicals. In this work, *Gloeophyllum trabeum* KU-41 was chosen for corn stover pretreatment through screening with 40 strains of wood-rot fungi. The objective of the current work is to find out which characteristics of corn stover pretreated with *G. trabeum* KU-41 determine the pretreatment method to be successful and worthwhile to apply. This will be done by determining the lignin content, structural carbohydrate, cellulose crystallinity, initial adsorption capacity of cellulase and specific surface area of pretreated corn stover.

**Results:**

The content of xylan in pretreated corn stover was decreased by 43% in comparison to the untreated corn stover. The initial cellulase adsorption capacity and the specific surface area of corn stover pretreated with *G. trabeum* were increased by 7.0- and 2.5-fold, respectively. Also there was little increase in the cellulose crystallinity of pretreated corn stover.

**Conclusion:**

*G. trabeum* has an efficient degradation system, and the results indicated that the conversion of cellulose to glucose increases as the accessibility of cellulose increases due to the partial removal of xylan and the structure breakage of the cell wall. This pretreatment method can be further explored as an alternative to the thermochemical pretreatment method.

## Background

Corn stover, the residue left in the field following the harvest of cereal grain, is a very common agricultural product in areas with high levels of corn production. The current annual production of corn stover is about 250 M tons in the U.S. [1] and 220 M tons in China [2]. Corn stover is in many ways an ideal feedstock for biomass ethanol production. Although biomass ethanol can be made from a wide range of biomass materials, stover from existing corn production is by far the most abundant crop residue readily available today.

Biomass ethanol is ethanol made from non-grain plant materials known as biomass. The bulk of most plants is fibrous material consisting of cellulose, hemicellulose and lignin. The bioconversion of biomass to ethanol requires hydrolysis of the carbohydrate polymers, cellulose changes to its constituent monomeric sugars prior to microbial fermentation. Due to the complicated structure of the cell wall in biomass, additional pretreatment is needed for biomass ethanol production compared to grain ethanol.

Biomass pretreatment is an essential step in biomass ethanol production with high yield [3,4]. Many studies have been published about different pretreatment methods for enhancing the digestibility of biomass [5,6]. Biological pretreatment that utilizes the metabolite of microorganism in nature to break up the cell wall of biomass for ethanol production is a promising technology due to its advantages of having a low energy requirement and being friendly to the environment [7,8]. Compared to chemical pretreatment, it is no need to recycle the chemical and does not bring exotic materials to environment. These reductions in the severity of pretreatment conditions could result in less biomass degradation and consequently lower inhibitor concentrations compared to conventional thermochemical pretreatment [5]. Fungal pretreatment using wood-rot fungus is one of the most effective methods for enhancing the efficiency of enzymatic saccharification [9]‐[12]. [Fissore et al 13] evaluated a process of combined brown-rot decay –chemical delignification as a pretreatment for bioethanol production. The combination of brown-rot fungi and organosolv processes result in 210 ml ethanol/ kg wood. Some thermochemical pretreatment methods have been performed for biomass ethanol production [4,14]‐[17]. However, biological pretreatment of corn stover with wood-rot fungi has been neglected. As is widely known, due to the ability to degrade lignin extensively, white-rot fungi (WRF) have received considerable attention for their potential to remove lignin for bio-ethanol pretreatment [8]. In contrast, brown-rot fungi (BRF) such as *Gloeophyllum trabeum*, have different mechanisms for the degradation of wood that rapidly depolymerize the cellulose and hemicellulose in wood with modified lignin in the brown residue. BRF degrade lignocellulose via a theorized two-part mechanism, with modification of the plant cell wall induced non-enzymatically and secretion of cellulases and hemicellulases likely occurring after modifications [18,19]. The initial stages of decay are thought to involve Fenton chemistry (Fe^2+^+H_2_O_2_) for the production of hydroxyl anions and radicals [20]. The low molecular weight reactants, unlike enzymes, are small enough to penetrate the wood lignocellulose fabric, and have been shown in immunolabeling studies to be present throughout the S2 layer of the brown rot-degraded cell wall [21]. Cellulase production by brown rot fungi is different in that it is typically constitutive, not influenced by free glucose concentrations, and most often lacks exo-acting cellobiohydrolase [22,23]. *G. trabeum* has the ability of fermenting sugar to ethanol [24]. The enzymatic and non-enzymatic mechanisms used by *G. trabeum* to degrade wood could potentially be employed for the bioconversion of other biomass, such as corn stover. The complicated structural modification of the cell wall plays a role in the initial degradation of BRF in the pretreatment for the purpose of bio-ethanol production.

In this study, 40 strains of wood-rot fungi (33 strains of WRF and 7 strains of BRF) were screened for corn stover pretreatment. A strain of brown-rot fungus KU-41 was selected for corn stover pretreatment due to its having the highest conversion of cellulose to glucose (CCG). A molecular biological identification showed that KU-41 was most closely related to *Gloeophyllum trabeum.* To gain a deeper understanding of the mechanisms of brown-rot fungus pretreatment, the fungal-pretreated corn stover was evaluated in detail, including the lignin and structural carbohydrate contents, cellulose crystallinity, initial adsorption capacity of cellulase, SEM and specific surface area.

## Results

### Fungal screening

**Figure 1:**
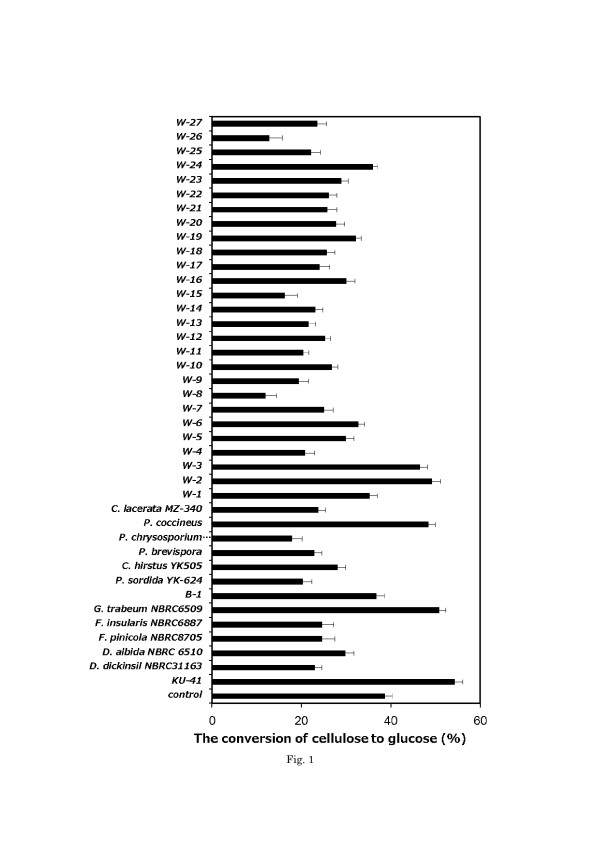
**CCG of corn stover pretreated with wood-rot fungi after 48 hours hydrolysis.** W means white-rot fungus unidentified; B means brown-rot fungus unidentified. CCG indicates glucose amount produced in the hydrolysis process/ cellulose amount in un-pretreated corn stover. Values are means ± SD of triplicate samples.

The CCG results of the pretreatment of corn stover with wood-rot fungi after 48 h of hydrolysis are shown in Figure 1. Compared to the control, the CCG levels of corn stover pretreated with most white-rot fungi were not increased except in those pretreated by *Pycnoporus coccineus*, W_2_ and W_3_, but this increase of CCG was not significant. We tried to use 7 strains of brown-rot fungi, which had the ability to decay wood significantly. The CCG of corn stover was not increased by pretreatment with these brown-rot fungi except for *G. trabeum* NBRC6509 and KU-41. In particular, the fungus KU-41 enhanced the CCG of corn stover. The CCG of pretreated corn stover was determined by the glucose yield and weight loss. Although some strains of wood-rot fungi have great degrading ability, they were not suitable for corn stover pretreatment due to the simultaneous degradation of cellulose. Of these 40 strains of wood-rot fungi, KU-41 caused the greatest increase in CCG. Therefore, KU-41 was selected for further study in order to evaluate its use in corn stover pretreatment.

### Fungal identification

Sequencing of the ITS rDNA region of KU-41 was performed, and the ITS sequence was submitted to Genbank with the accession number JF682770-JF682771. The strain showed the highest identity (99%) with *G. trabeum*. Based on the comparison of the ITS rDNA gene sequences, the strain chosen for corn stover pretreatment was identified as a strain of *G. trabeum* and was named *G. trabeum* KU-41. We chose another strain of *G. trabeum* NBRC6430 to pretreat the corn stover, and compared the efficiency of this pretreatment to that of *Gtrabeum* KU-41.

### Pretreatment conditions and enzymatic hydrolysis

**Figure 2:**
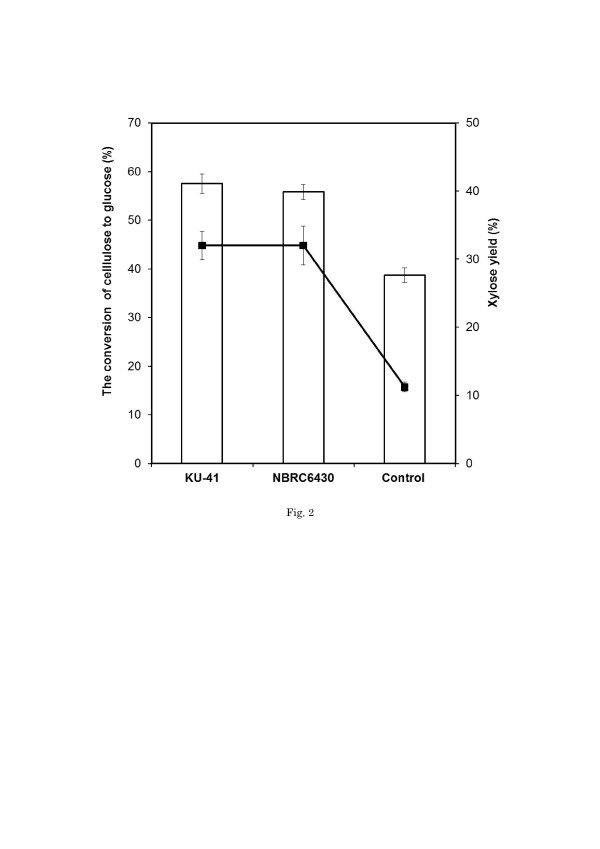
**CCG and xylose yield of pretreated corn stover after 48 hours hydrolysis.** Bar expresses CCG; line expresses xylose yield. Xylose yield means xylose amount produced during hydrolysis/xylose released by totally acid hydrolysis in pretreated corn stover. Values are means ± SD of triplicate samples.

In the process of corn stover pretreatment with wood-rot fungi, moisture content and pretreatment period were important factors that affected the efficiency of pretreatment. The effect of moisture content on the pretreatment period and the CCG of corn stover pretreated with KU-41 was tested (data not shown). The highest CCG was obtained after 20-day pretreatment with 80% moisture content. The efficiency of pretreatment was evaluated by enzymatic hydrolysis. Figure 2 shows enzymatic hydrolysis of the samples after 48 h. As expected, corn stover without pretreatment was capable of converting cellulose into glucose with an efficiency of 38.7%. High glucose yields were obtained when the corn stover was pretreated by KU-41 and NBRC6430. The CCG levels increased by 47% and 42% over 20-day treatment with KU-41 and NBRC6430,respectively. The strain of *G. trabeum* KU-41 seemed to be the most promising fungus for biological pretreatment of corn stover.

Hemicellulose serves as a connection between lignin and cellulose fibrils and gives the whole cellulose-hemicellulose-lignin network more rigidity [25]. Xylan is the main component of hemicellulose in corn stover. In corn stover pretreated with KU-41 and NBRC6430, 32.0% and 31.4% xylan was transformed into xylose and released after 48 h of hydrolysis, in comparison with only 11.2% xylan transformation in the un-pretreated sample (Figure 2). The xylose yield in the saccharification solution of pretreated corn stover was 2.8-fold greater than that of the un-pretreated sample suggested that during corn stover pretreatment with *G. trabeum*, the hemicellulose structure modification or breakage is beneficial for xylose release during enzymatic hydrolysis. An increase in xylose release led to an increase in cellulose accessibility, which consequently resulted in the glucose yield increasing in corn stover pretreated by *G. trabeum* KU-41and *G. trabeum* NBRC6430*.*

### Compositional analysis

Compositional analysis of corn stover pretreated with fungus (Table 1) gave a good indication of the possible changes in the different components. As the pretreatment progressed, hemicellulose was the component that was removed in the greatest proportion. Biodegradation of hemicellulose from corn stover was 43% with *G. trabeum* KU-41 and 43% with *G. trabeum* NBRC6430. As hemicellulose is connected with cellulose microfibrils, the prior removal of these can facilitate cellulose degradation. The content of glucan decreased slightly, reaching removal percentages of 16.8% and 18.3% with *G. trabeum* KU-41 and *G. trabeum* NBRC6430, respectively. Consistent with the degradation mechanism of brown-rot fungi, lignin was essentially undegraded by the two strains of *G. trabeum*.

**Table 1:** Main composition of unpretreated and pretreated corn stover and ratio of component left after pretreatment

**Pretreatment**	**Composition of pretreated corn stover (%)**			**Ratio of component left in pretreated corn stover (%)**		
	**G**	**X**	**L + A**	**G**	**X**	**L + A**
*G. trabeum* KU-41	39.1	12.0	33.0	83.2	57.0	96.1
*G. trabeum* NBRC 6430	38.4	12.0	33.3	81.7	57.0	97.0
Control	35.7	16.0	26.1	100.0	100.0	100.0

G: glucan.

X: xylan.

L + A: total lignin + ash.

### Crystallinity determination

Lignocellulosic biomass is mainly composed of cellulose (amorphous and crystalline), hemicellulose and lignin. Crystallinity is believed to affect enzymatic saccharification of cellulose [25]. However, due to the complex structure of the cell wall in lignocellulosic biomass, it is impossible to separate cellulose from other components completely or to measure the cellulose crystallinity directly. X-ray measurement of CrI is still the most frequently-used method to estimate the effect of pretreatment on biomass crystallinity. The CrI values of pretreated corn stover samples here were determined by measuring the relative amount of crystalline cellulose in the total solid. The cellulose crystallinity (CrI %) in corn stover pretreated with *G. trabeum* KU-41(55.3 ± 0.02) and *G. trabeum* NBRC6430 (55 ± 1.37) was slightly increased compared to the control (53.9 ± 2.01). The slight increase of CrI in the pretreated samples suggested that the cellulose became more exposed after pretreatment. However, all materials give rise to X-ray scattering, with the amorphous part including not only amorphous cellulose but also hemicellulose and lignin. Hemicellulose and lignin have been determined to have diffractograms similar to amorphous cellulose [26], giving wide unspecific peaks, which may affect the results.

### Initial adsorption capacity

**Figure 3:**
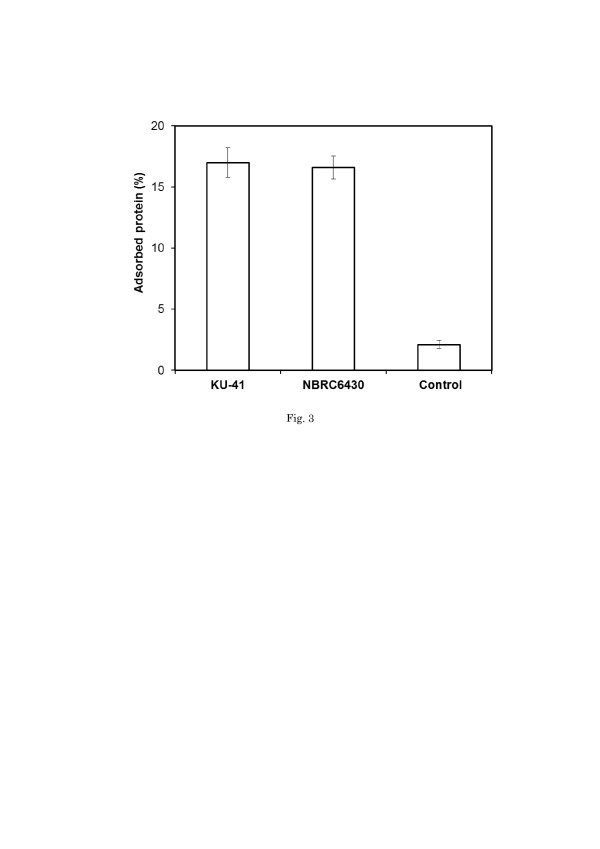
**Initial adsorption capacity of cellulase onto corn stover after pretreatment with *G. trabeum* .** Values are means ± SD of triplicate samples.

[Jeoh et al. 27] claimed that the hydrolysis rate and/or yield is directly related to the amount of adsorbed enzymes. Figure 3 shows the initial adsorption capacity of cellulase onto corn stover after pretreatment with *G. trabeum*. The pretreated corn stover showed a significant increase of the initial adsorption capacity. The initial adsorption capacity increased about 7-fold after pretreatment with *G. trabeum*. Lignin is believed to impede enzyme access to glucan chains by its binding and steric hindrance [28,29]. In this study, the lignin ratio in corn stover pretreated with *G. trabeum* KU-41 increased by 30% (Table 1), while the CCG was enhanced by 46% (Figure 2). Evidently, the lignin content is not the main reason of the increase of the cellulase initial adsorption capacity in corn stover pretreated with *G. trabeum.*[Kumar and Wyman 6] proposed that lignin did not directly control the cellulose accessibility but restricted the xylan accessibility, which in turn controlled access to cellulose. The xylose yield of pretreated corn stover increased 2.8-fold (Figure 2). So we have reason to believe that the relationship among lignin, hemicellulose and cellulose changed such that the lignin influence was weakened in cellulose hydrolysis during the process of corn stover pretreatment with *G. trabeum* KU-41 and *G. trabeum* NBRC6430.

### Microstructure analysis

**Figure 4:**
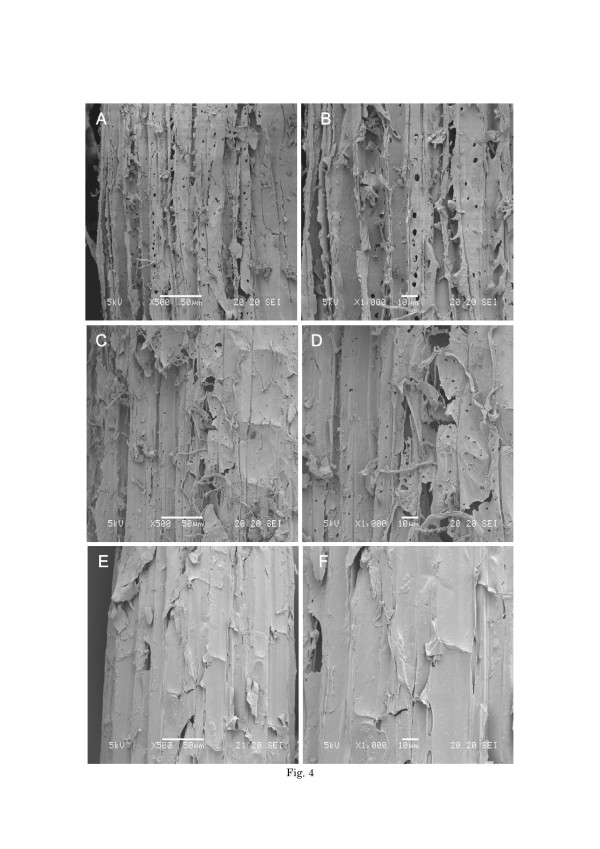
**SEM images of un-pretreated and pretreated corn stover samples with *G. trabeum* .[A-B]** pretreated sample with *G. trabeum* KU-41 500x **(A)**, 1000x **(B)**; **[C-D]** pretreated sample with *G. trabeum* NBRC 6430 500x **(C)**, 1000x **(D)**; **[E-F]** un-pretreated sample 500x **(E)**, 1000x **(F)**.

SEM images of corn stover pretreated with fungus are shown in Figure 4 at magnifications of 500 and 1000. The surface of the untreated corn stover was compact and rigid, suggesting that this property hinders the accessibility of cellulase to cellulose (Figure 4E-F). More pores and cracks were created on the surface of the corn stover (Figure 4A-B, 4 C-D), and fungal pretreatment seemed to disrupt the biomass structure to some extent.

**Figure 5:**
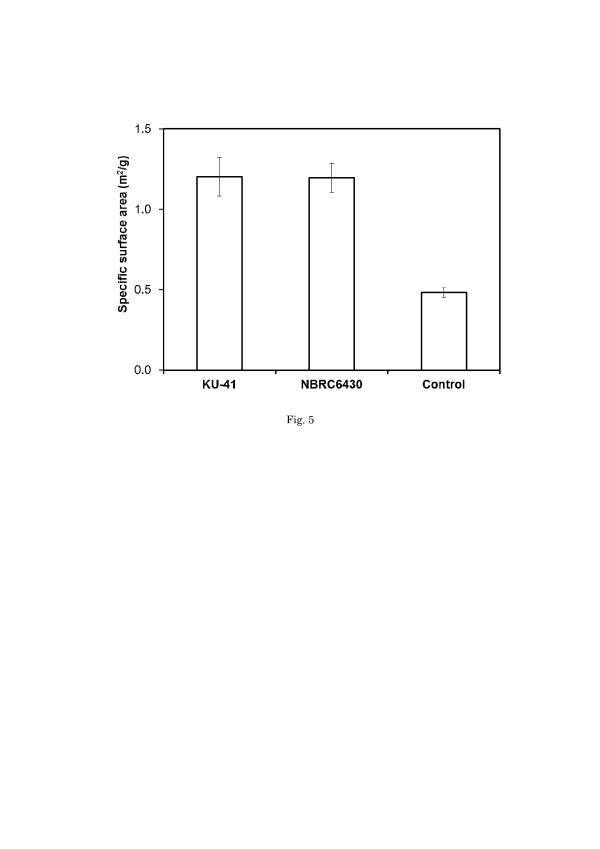
**Specific surface area of corn stover pretreated and un-pretreated with *G. trabeum* .** Values are means ± SD of triplicate samples.

The specific surface area and pore distribution are generally considered to play important roles in accessibility. It has been reported that the cellulase digestibility of pretreated biomass is limited by cellulose accessibility [27]. Here (Figure 5), a significant difference in the specific area between the control value and that of pretreatment samples was determined. The specific surface of corn stover pretreated with *G. trabeum* was increased 2.5-fold.

Based on comprehensive analysis of Figures 4 and 5, the SEM demonstrated that the fungal pretreatment could cause severe degradation and evident damage to the intact cell structure. Both the selective biodegradation of cellulose and hemicellulose components by the fungus and fungal penetration might contribute to the pore production. Removal of hemicellulose increased the mean pore size of the substrate and therefore increased the probability of the cellulose to be hydrolyzed [30,31]. Drying of the pretreated lignocellulose can cause a collapse in pore structure, resulting in decreased enzymatic hydrolysis [32]. Cellulosic particles have both external and internal surfaces. The internal surface area of porous cellulose particles depends on the capillary structure and includes intraparticulate pores (1–10 nm) as well as interparticulate voids [33]. External surface area is closely related to shape and particle size, and can be estimated by microscopic observation [34]. [Zhang and Lynd 35] suggested that cellulase can get trapped in the pores if the internal area is much larger than the external area, which is the case for much lignocellulosic biomass. Pore production, which was a result of the pretreatment, led to a significant increase in specific surface area. These phenomena are probably the reasons that the CCG of corn stover pretreated with *G. trabeum* was enhanced compared to the control.

## Discussion

Fungal pretreatment followed by thermochemical pretreatment could potentially lower the severity requirements of acid, temperature and pressure in thermochemical pretreatment [5]. Lower pretreatment severity is expected to translate directly into lower chemical consumption, and because of lower temperature requirements, possibly lower steam cost. These reductions in severity could also reduce the capital costs and result in lower biomass degradation and consequently lower inhibitor levels. This potential of fungal pretreatment can be explained by the ability of certain fungi to disrupt the structure of the cell wall, resulting in partial breakdown of the lignin/carbohydrate complex. Lignin is a complex aromatic polymer based on phenylpropane units. It contains few hydrolysable bonds and so it is poorly susceptible to attack by hydrolytic enzymes. Lignin and its associated compounds have been found to inhibit cellulase action [36]. There is evidence that lignin may retard cellulase action by adsorbing cellulase enzyme [37]. Delignification is a method of increasing the biomass susceptibility of hydrolysis. However, some papers reported that attempts to correlate the delignification of biomass to its increasing susceptibility to enzyme digestion were often inconclusive or contradictory depending on variables such as the type of substrate and the pretreatment conditions [38]‐[40]. [Stranks 41] has explained why the lignin-holocellulose relationship rather than the lignin concentration was responsible for the effect of the lignin on cellulose saccharification. [Wong et al 42] have suggested that lignin distribution maybe as important as accessibility in determining the enzymatic digestibility of pretreated fibers by steam explosion. [Gharpuray et al. 43] also discussed the probable interdependent effects of lignin concentration and surface area on hydrolysis. The complex physical relationship between lignin and cellulose fibrils revealed by [Ruel et al. 44] also supports the proposition that lignin distribution is more important to enzymatic hydrolysis than lignin concentration. [Yelle et al. 45] reported that brown-rot of spruce wood by *Gloeophyllum trabeum* resulted in a marked, non-selective depletion of all intermonomer side-chain linkages in the lignin. As shown in Table 1, the lignin ratio in corn stover pretreated with *G. trabeum* was increased compared to the control. But the CCG (Figure 2) in corn stover pretreated with *G. trabeum* was enhanced significantly. We believe that the structure of lignin in the process of fungal pretreatment was modified despite the fact that amount was not decreased. The bonds between cellulose and lignin or hemicellulose and lignin were modified or broken [46], which may be one reason that the CCG in corn stover pretreated with *G. trabeum* KU-41 was enhanced.

The association between the cellulose components and the hemicellulose appears to be of a physical nature, as there have been no reports of chemical bonds between cellulose and hemicellulose molecules. In a series of papers [37,47,48] on the effects of various pretreatments on enzymatic hydrolysis, it was found that in pretreatments which removed only lignin, the rate of digestion of the hemicellulosic component was similar to that of cellulose. This result suggested that the relationship between cellulose and hemicellulose was that one must be removed to facilitate an attack on the other. Increases in cellulose digestibility have been associated with decreased hemicellulose content; more than 80% of hemicellulose was degraded by a dilute sulfuric acid pretreatment [4]. In this study, 43% hemicellulose degradation was observed. One interpretation of these data is that removing xylan during pretreatment facilitated cellulase access to the cellulose, which resulted in increased digestibility. This concept has been discussed in the literature as relating changes in the porosity of the biomass sample to digestibility [49]. We believe that the removal of lignin or hemicellulose or both serves one purpose: disruption of carbohydrate-lignin networking, which enhances enzyme adsorption, generally labeled as accessibility.

With lignocellulosic materials, crystallinity appears to be of lesser importance than the association between lignin (and hemicellulose) and surface area. Pretreatments such as ball milling reduced crystallinity but did not have a corresponding effect on hydrolysis [43]. When cardboard and newspaper were used, a decrease in crystallinity resulted in a significant increase in saccharification [50]. With cryomilled rice hulls, the relationship was not apparent [51], while alkali treatment of rice straw resulted in an increase in the crystallinity index from 40 to 52% and an increase in solubilization from 12 to 63% [52]. It was also found that the crystallinity of cellulose in wood was not an inhibitory factor in their enzymatic hydrolysis experiments [53]. The effects of various chemical pretreatments on a range of substrates have resulted in no change or an actual increase in crystallinity after the pretreatment, while the rate and extent of enzymatic hydrolysis were markedly increased. [Puri 54] concluded that any increase was probably due to the removal of amorphous material rather than any increase in the ordered region and that crystallinity was not as important a factor as generally believed. In this study, Avicel [(R) RH-101 (Sigma)] was used as the sole carbohydrate for cultivating *G. trabeum* KU-41; the fungus grew very slowly and the crystallinity of Avicel did not change (data not shown). This result was consistent with the finding that most brown-rot fungi showed slow growth on crystalline cellulose despite their rapid degradation of cellulose in wood [55]. The present results indicate that the fungal pretreatment cleavages cellulose in the noncrystalline regions. Part of the amorphous cellulose degradation led to a change of the cellulose structure, which increased the surface area with access to cellulase in pretreated corn stover.

## Conclusion

Although biotechnology presents important opportunities for achieving very low costs, pretreatment of naturally resistant cellulosic materials is essential if we are to achieve high yields from enzymatic hydrolysis. Fungus pretreatment with *G. trabeum* proved to be an effective way of increasing enzymatic hydrolysis of corn stover for bio-ethanol production. As is widely known, pretreatment with white-rot fungi could increase the enzymatic hydrolysis of biomass through lignin degradation. What happened in the process of pretreatment with brown-rot fungus is the crux of this paper. The results showed that the pretreatment by *G. trabeum* had a partial defibrating effect on corn stover. Partial removal of xylan and modification of the structure of lignin resulted in disrupting the structure of the cell wall. The disruption of the structure of the cell wall increased the accessibility of cellulase to lignocellulose.

A future goal is to improve the efficiency of the fungus pretreatment method described in this study by substantially reducing the cost and accelerating its commercial application. One approach that is attracting attention is the pretreatment of corn stover in vitro with the goal of a short pretreatment time. We believe that with further research and the development of technology, fungus pretreatment could achieve a real decrease in the cost of bio-ethanol production.

## Methods

### Fungal stains and isolation

We selected 33 strains of WRF and 7 strains of BRW that decay wood significantly for use in corn stover pretreatment. *Daedalea albida* NBRC6510, *Daedalea dickinsii* NBRC31163, *Fomitopsis insularis* NBRC6887, *Fomitopsis pinicola* NBRC8705, *Gloeophyllum trabeum* NBRC6509 and *Phlebia brevispora* TMIC33929 were obtained from the Biological Resource Center (Chiba, Japan) and Tottori Mycological Institute (Tottori, Japan). *Coriolus hirstus* YK-505 [56], *Ceriporia lacelate* MZ-340 and *Phanerochaete sordida* YK-624 [57] were obtained from stock cultures in our laboratory. KU-41 and other strains were isolated from decayed wood in a cedar forest at Morotuka, Higashiusuki district, Miyazaki, Japan. Stock cultures of the fungi were maintained on potato dextrose agar (PDA, Difco, Detroit, MI, USA) plates at 4°C.

### Preparation of corn stover

Corn stover was collected from Yingkou city in Liaoning Province in China. The stover was then chopped air-dried and stored at room temperature. The constituents of corn stover (lignin: 23.3%, hemicellulose: 28.1%, cellulose: 35.4%, cell solubles: 12.4%, ash: 2.8%) were determined by following a detergent digestion protocol [58]. Before pretreatment, the corn stover was milled and ground to 250–350 μm in size, cleaned with water to remove the soluble content and then filtered, freeze-dried and subjected to treatment with wood-rot fungi.

### Fungal screening

The mycelium was transferred to a new PDA medium in a 9-cm diameter Petri dish and incubated at 30°C. Once the fungus covered most of the PDA plate, agar plates with mycelium were transferred to a sterile blender cup containing 25 ml of sterile water and homogenized for 30 s. one milliliter of the white-rot fungus homogenate was used to inoculate 10 ml of low-nitrogen basal III medium [59] and brown-rot fungus homogenate was inoculated into 10 ml potato dextrose broth (PDB) medium in 100-ml Erlenmeyer flasks. When the fungi covered the medium surface, the mixture was vortexed with 25 ml sterile water to mix it well, and 1 ml was inoculated into 5 g corn stover powder and then cultivated at 30°C for 30 days [5]. Prior to fungal inoculation, the corn stover was sterilized in the autoclave for 20 min at 121°C, and then 5 ml of sterilized water was added to the corn stover to keep the moisture content at 50%. Corn stover treated in the same conditions without fungal inoculation was used as the control. After pretreatment, the corn stover was washed to remove the soluble content with 250 ml water, then filtered and freeze-dried. Weight loss was estimated as the difference between the weight of the corn stover at the beginning and at the end of the pretreatment according to the following formula: weight loss (%) = (W_1_-W_2_)/W_1_×100, where W_1_ is the weight of the sample before pretreatment, and W_2_ represents the weight of the sample after pretreatment. All experiments were performed in triplicate.

### Saccharification

CCG was conducted to estimate the efficiency of the bio-pretreatment. Corn stover without pretreatment was used as the control. A 1 g corn stover sample with 0.7 g cellulase T3 (HBI Enzymes Inc. cellulase activity: 240 FPU/g; xylanase activity: 63.62 U/g) was added into 50 ml of 0.1 M sodium acetate buffer (pH 4.6) and incubated with gentle shaking (100 rpm) at 60°C. After 48 h, the release of glucose was determined by a Biosensor BF-5 (Oji Scientific Instruments Co., Itd). All experiments were performed in triplicate. Upon evaluation of the effects of pretreatment, CCG was defined as the percentage of cellulose in the raw material converted to glucose, taking into account the weight loss during pretreatment.

### Identification of microorganism

The ITS1-5.8-ITS2 ribosomal RNA gene of KU-41 was amplified by PCR using the primer set ITS1 primer (5'-TCCGTAGGTGAACCTGCGG-3') and ITS4 primer (5'-TCCTCCGCTTATTGATATGC-3'). The 626-bp amplicon obtained was cloned and sequenced. The sequences were proofread, edited, and merged into composite sequences using Clustalx-1.83.1. The fungus was determined to be most closely related to *Gloeophyllum trabeum* by comparing it with related strains in GenBank. The GenBank accession numbers of KU-41 are JF682770-JF682771.

### Corn stover pretreatment

Another strain of *G. trabeum* NBRC6430 obtained from the Biological Resource Center (Chiba, Japan) was chosen to compare with the efficiency of corn stover pretreatment with *G. trabeum* KU-41. The biological pretreatment with *G. trabeum* KU-41 was carried out in 250 ml Erlenmeyer flasks with 10 g corn stover powder and 40 ml distilled water (80% moisture content) for a 20-day pretreatment. The other conditions were the same as described in the fungal screening. The release of glucose was determined by a Biosensor BF-5. The xylose from enzyme hydrolysis was determined by HPLC (HITACHI, RI detector, Shodex Asahipak NH2P-50 4E column; eluent: CH_3_CN/H_2_O = 75/25; flow rate: 1 ml/min; temperature: 30°C).

### Chemical component analysis

The lignin composition in corn stover pretreated with *G. trabeum* was determined by the method that determination of structural carbohydrates and lignin in biomass published by the National Renewable Energy Laboratory [60]. The carbohydrate composition was determined by total acidic hydrolysis followed by analysis of monomeric sugars by GC-MS [61]. Corn stover pretreated by *G. trabeum* was milled and then subjected to acid hydrolysis. A 300 mg sample of pretreated corn stover was hydrolyzed with 72% sulfuric acid at 30°C for 4 h. The secondary hydrolysis was performed after dilution with water to a 3% sulfuric acid concentration by autoclaving at 121°C for 60 min. Then the hydrolysate was filtered through a glass filter. The acidic filtrate containing sugars was adjusted to a pH of 5–6 with barium hydroxide. The reduced monomeric sugars with NaBH_4_ were acetylated and then analyzed using a Shimadzu gas chromatography-mass spectrometer (GC-17A and GCMS-QP5050) with a glass capillary column, DB-5. The temperature program: 140°C for 3 min; 140°C-280°C, at a rate of 6°C /min; 280°C-320°C, at a rate of 10°C /min; 320°C for 5 min.

### Cellulose crystallinity

The crystallinity of the pretreated corn stover powder was measured by high resolution X-ray diffractometry (XD-D1 X-Ray diffractometer, Shimadzu, Japan). The measurement conditions were 40 kV and 40 mA. There were triplicate samples a_1_, a_2_, and a_3_ pretreated with the same fungus, respectively. Each sample was scanned from 2θ = 5° to 35° with a step size of 0.05 and tested three times to get the mean values ā. Crystallinity (%) was defined as [(I_002_ − I_am_)/I_002_] × 100%, where I_002_ and I_am_ are the maximum intensity at 2 θ = 22.6° and the minimum intensity at 2θ = 18.7°, respectively. The crystallinity value of corn stover pretreated with certain strain of fungus is mean ± SD of ā_1_, ā_2_ and ā_3_.

### Initial adsorption capacity of cellulase

A total of 0.21 g of commercial cellulase (Cellulosin T3, HBI Enzymes Inc.) was added to 0.3 g corn stover pretreated with *G. trabeum* KU-41 and *G. trabeum* NBRC6430. The corn stover pretreated with fungus without the addition of cellulase was used for the control. The mixture was added to 15 ml, 100 mM sodium acetate buffer (pH 4.6). These reactions were placed at 4°C for 90 min with 100 rpm to reach adsorption equilibrium and were stopped by centrifugation at 8000 rpm for 20 min. Protein in the supernatant was measured by using the Bradford protein assay (Bio-Rad, Hercules, California, USA). Adsorbed cellulase was determined as the difference between the amount of protein initially added and the amount of un-adsorbed protein in the supernatant.

### Scanning electron microscopy (SEM)

SEM pictures of untreated and pretreated corn stover (dried powder of particle size between 250 μm and 300 μm) were taken at magnifications of 500 times using a JSM-5600LV1 scanning electron microscope at 5 kV. Prior to taking the pictures, the samples were sputter- coated with a thin layer of gold.

### Specific surface area determination

The specific surface area of the pretreated corn stover sample was estimated by the Brunauer-Emmett-Teller (BET) method [62].

## Competing interests

The authors declare that they have no competing interests.

## Authors’ contributions

ZG participated in the design of the study, performed all the laboratory scale experiments and took part in the interpretation of the results and the writing of the manuscript. TM carried out fungal identification and took part in the interpretation of the results. RK participated in the design of the study, took part in the interpretation of the results, and was the main author. All authors suggested modification to the draft, commented on several preliminary versions of the text, and approved the final manuscript. All authors read and approved the final manuscript.
